# School-Age Neurodevelopmental and Atopy Outcomes in Extremely Preterm Infants: Follow-Up from the Single Versus Triple-Strain Bifidobacterium Randomized Controlled Trial

**DOI:** 10.3390/nu18010141

**Published:** 2026-01-01

**Authors:** Gayatri Athalye-Jape, Chandra Rath, Meera Esvaran, Angela Jacques, Sanjay Patole

**Affiliations:** 1 Neonatology, King Edward Memorial Hospital, Child and Adolescent Health Service, Perth, WA 6008, Australia; drcprath@gmail.com (C.R.); sanjay.patole@health.wa.gov.au (S.P.); 2 School of Medicine, The University of Western Australia, Perth, WA 6009, Australia; 3 Neonatology, Canberra Hospital, Canberra, ACT 2605, Australia; 4 Centre for Marine Science and Innovation, University of New South Wales, Sydney, NSW 2052, Australia; meera.esvaran@gmail.com; 5 Research Department, Perth Children’s Hospital, Child and Adolescent Health Service, Nedlands, WA 6009, Australia; angela.jacques@health.wa.gov.au

**Keywords:** probiotic, preterm, neurodevelopment, neurobehavior, atopy, gut brain axis, Bifidobacterium, extreme preterm

## Abstract

**Background:** Probiotic supplementation for very preterm infants is a common practice in many neonatal units. Assessing the effects of early postnatal exposure to probiotics on long-term neurodevelopment, growth, and atopy-related outcomes is important. Extremely preterm (EP: <28 weeks) infants enrolled in our previously reported randomized trial (SiMPro) comparing short-term effects of single (SS: B. breve M-16V) versus triple-strain (TS: B. breve M-16V, B. longum subsp. infantis-M63, B. longum subsp. longum-BB536) probiotic provided a unique opportunity to study this issue. **Methods:** This follow-up study assessed the five-year outcomes of SiMPro trial infants, including neurodevelopment (cognition (Full Scale Intelligence Quotient/ FSIQ using WPPSI-IV), behavior (Strengths and Difficulties Questionnaire), executive function (BRIEF–P)), growth (anthropometry) and blood pressure (BP). Atopy-related outcomes were evaluated at six to seven years using the ISAAC questionnaire. A linear mixed model was used for longitudinal outcomes. Impairment indicators were modeled using logistic regression and adjusted for Socio-Economic Indexes for Areas (SEIFA) centiles. **Results:** Follow-up rates (SS: 89.2% versus TS: 95%), neurodevelopmental outcomes [severe impairment (FSIQ < 70): SS: 7.4% versus TS: 4.3%; *p* = 0.68], growth, BMI, and BP were comparable between the SS and TS groups. The total difficulty score or BRIEF–P executive indices, disability rates (none: 66.7% versus 55.4%), and atopy-related outcomes were comparable between groups. **Conclusions:** Both TS and SS Bifidobacterium probiotic formulations were safe, with comparable neurodevelopmental, growth, and atopy-related outcomes at school age.

## 1. Introduction

Extremely preterm (EP; gestation < 28 weeks) infants are at higher risk for adverse neurodevelopmental outcomes, including impaired cognition, motor function, language, and socio-emotional regulation [1,2,3]. This vulnerability relates to the complex interplay of perinatal brain injury, systemic inflammation, an immature immune system and dysbiosis during a critical period of rapid brain development [4,5,6,7]. Increasing evidence implicates the gut–microbiome–brain axis as a key mediator of neurodevelopment, modulating neuronal maturation, synaptic plasticity, and neuroimmune interactions [8,9,10].

Probiotic supplementation is a promising strategy to optimize neurodevelopment in preterm infants. Many pathways have been proposed to explain the potential benefits of probiotics in this context, including their anti-inflammatory and immunomodulatory effect and ability to counteract dysbiosis. It is important to note that systemic inflammation and gut dysbiosis are strongly linked to white matter injury and delayed cortical maturation in preterm infants [5,11,12]. Short-chain fatty acids (SCFAs) produced by commensal microbes are known to enhance the gut-brain barrier and modulate microglial activity and neurotransmitter synthesis, providing a mechanistic link between microbial modulation and neurodevelopmental outcomes [13,14,15,16,17].

Gut microbiota composition and function during infancy, especially Bifidobacterial predominance, are associated with later life cognitive outcomes [18,19]. Longitudinal cohort studies have reported an association of early Bifidobacterium predominance with improved neurodevelopment and an association of dysbiosis with adverse neurodevelopmental outcomes [20,21]. Probiotics, particularly Bifidobacterium and Lactobacillus species, are known to enhance intestinal barrier integrity, attenuate inflammatory cytokine responses, and generate neuroactive metabolites [13,14,15,16,17]. Probiotic strains such as *B. longum* subsp. *infantis* can metabolize human milk oligosaccharides into indole-3-lactic acid and tryptophan derivatives that exert immunoregulatory and neuroactive effects by modulating various signalling pathways and one-carbon energy metabolism essential for myelination [22,23,24,25]. Such microbial–metabolic interactions provide a plausible pathway through which probiotics may optimize neurodevelopmental maturation and function during early childhood [26,27]. Additionally, an early-life microbiota with increased Bifidobacteria and human milk oligosaccharide (HMO) utilization genes has been linked to reduced risk of childhood allergic disease [28].

Probiotic supplementation for very preterm infants is a common practice in many neonatal units. Hence, assessing the effects of early postnatal life exposure to probiotics on long-term neurodevelopment, growth, and atopy-related outcomes is important in this cohort. Extremely preterm (EP: <28 weeks) infants enrolled in our previously reported randomized trial (SiMPro) comparing short-term effects of single (SS: B. *breve* M-16V; *n* = 86) versus triple-strain (TS: B. *breve* M-16V, B. *longum subsp. infantis*-M63, B. *longum subsp. longum*-BB536, *n* = 87) probiotic provided a unique opportunity to study this issue. The primary outcome of this trial, time to full enteral feeds (150 mL/kg/day), did not differ significantly between groups. However, both probiotic regimens effectively reduced dysbiosis, marked by increased *Bifidobacterium* abundance and decreased *Gammaproteobacteria* [29].

This follow-up study aimed to assess the long-term (five to seven years) neurodevelopment, growth, and atopy-related outcomes of SiMPro trial infants.

## 2. Materials and Methods

The SiMPro RCT enrolled EP neonates born <28 weeks’ gestation who were ready to commence feeds or on feeds for <12 h. The methodological details of SiMPro RCT are reported in the original publication [29]. The primary outcome of the current prospective follow-up study was to assess the five-year neurodevelopmental outcomes of the SiMPro trial cohort. Secondary outcomes included neurobehavior, growth, and blood pressure (BP) at five years and atopy-related outcomes at six to seven years of age. All outcome assessors were blinded to the treatment (i.e., SS or TS probiotic) groups.

### 2.1. Neurodevelopmental and Neurobehavioral Outcomes

Standardized developmental assessments at five years included the Wechsler Preschool and Primary Scale of Intelligence, 4th Edition (WPPSI-IV), together with neurobehavioral profiling based on parent-report instruments. The WPPSI-IV was selected based on its broad international validation, predictive utility for school readiness and later academic achievement, and an established role in preterm population surveillance programs, including the neonatal developmental follow-up program in our hospital [30,31]. The WPPSI yields composite scores for verbal comprehension, visuospatial processing, fluid reasoning, processing speed, working memory, and full-scale IQ (FSIQ). It has demonstrated predictive validity for identifying cognitive deficit and correlates strongly with later measures of academic functioning and neuropsychological outcomes, supporting its use in early school-age follow-up of children born preterm [32].

Neurobehavioral outcomes were assessed using the Strengths and Difficulties Questionnaire (SDQ), selected for its strong global validation, diagnostic sensitivity, and demonstrated ability to detect socio-emotional strengths and difficulties across culturally and developmentally diverse populations of children [33,34]. SDQ is widely used in epidemiological and clinical research, enabling comparability with existing preterm and neurodevelopmental cohorts. Executive function was evaluated using the Behavior Rating Inventory of Executive Function–Preschool Version (BRIEF–P), a tool with robust reliability and validity for measuring inhibitory control, cognitive flexibility, and related executive domains in children with and without neurodevelopmental disorders. The BRIEF–P’s established clinical threshold (T-score ≥ 65) reliably identifies elevated executive dysfunction and supports its use in longitudinal follow-up studies [35,36,37].

Cerebral Palsy (CP) was defined clinically as a non-progressive disorder of movement and posture in the presence of tone abnormalities and, where possible, was classified under the Gross Motor Functional Classification System (GMFCS). CP status was verified against the West Australian Register of Developmental Anomalies–Cerebral Palsy register (WARDA–CP). Disability was defined as a pre-defined criterion [38] (Table 1).

Those who did not present for a formal assessment were evaluated via telehealth using the 60-month ages and stages questionnaire (https://agesandstages.com; accessed on 30 September 2022) and assigned a disability level. SDQ and BRIEF–P were not offered during telehealth for pragmatic purposes.

### 2.2. Growth, Blood Pressure, and Atopy Related Outcomes

Anthropometric data, including weight, length/height, and head circumference, were collected at follow-up visits at five years and interpreted using WHO Child Growth Standards to generate age- and sex-adjusted z-scores [39]. Body mass index (BMI) and BMI z-scores were calculated accordingly. BP was routinely measured at five years, recognizing the elevated cardiometabolic risk within preterm-born children [40].

Atopy-related outcomes were evaluated at six to seven years via the International Study of Asthma and Allergies in Childhood (ISAAC) questionnaire (https://isaac.auckland.ac.nz/resources/tools.php; ISAAC Tools accessed on 24 April 2023). The ISAAC tool has demonstrated robust validity for assessing atopy-related outcomes in childhood cohorts and facilitated international comparison [41,42].

### 2.3. Statistical Analysis for Clinical Data

Descriptive summaries included frequency distributions for categorical data or medians and interquartile ranges (IQR) for continuous data. Univariate comparisons between treatment groups were carried out using Chi-squared and Mann–Whitney U tests for categorical and continuous data, respectively. Bonferroni correction procedures were applied to account for the number of comparisons performed. Impairment indicators were modeled using logistic regression and adjusted for Socio-Economic Indexes for Areas (SEIFA) centiles, with results summarized with odds ratios (OR) and adjusted OR with 95% CI. Linearity of the outcome log odds was assessed by using the Box-Tidwell approach. Case-wise diagnostics were used to assess data for outliers. Alpha was set at 0.05, and all analyses were two-sided. Stata version 19 (StataCorp, College Station, TX, USA) was used for data analysis.

## 3. Results

Randomization in the SiMPro RCT was associated with no statistically significant differences in baseline demographic variables, including sex, gestational age, and socioeconomic status, between the SS and TS probiotic groups, minimizing the risk of confounding (Table 2). Socioeconomic indices, specifically SEIFA, indicated cohort representativeness. Data for parental education were missing for 77% of cases.

### 3.1. Neurodevelopmental and Neurobehavioral Outcomes

**Figure 1:**
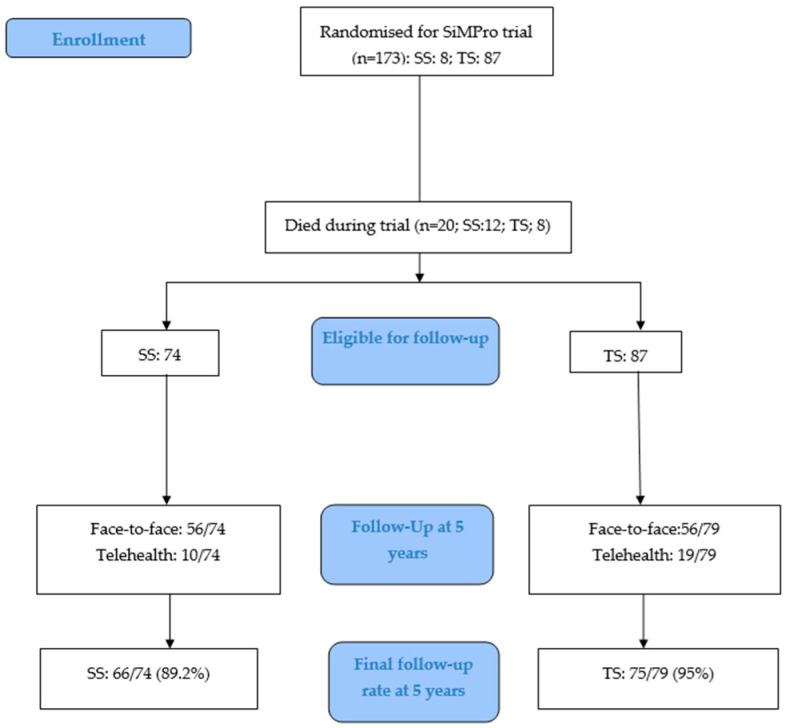
Flow diagram showing participant follow-up.

A total of 20 participants died during the original RCT (SS: 12 and TS: 8) [31] thus, survivors who were eligible for follow-up (FU) were SS: 74 and TS: 79. FU rates (face-to-face: SS: 56/74; TS: 56/79 and telehealth: SS: 10/74; TS: 19/79) at five years (SS: 89.2% versus TS: 95%) were comparable between the two groups of the trial (Figure 1).

Prevalence and severity of CP, autism, blindness, and deafness at school age were comparable between SS and TS groups, with no statistically significant differences observed in any outcome category. Similarly, no significant difference was noted SDQ and BRIEF-T scores between the groups. (Table 3).

At school age, no statistically significant differences were observed between the two groups across severity levels (Table 4).

### 3.2. Growth Outcomes

At school age, there were no statistically significant differences between SS and TS probiotic groups in height, weight, or head circumference, with mean values and z-scores closely matched across all anthropometric measures (Table 5).

### 3.3. Blood Pressure and Atopy-Related Outcomes

At five years, BMI, BP, and atopy-related outcomes remained similar (Table 6).

## 4. Discussion

Our prospective long-term follow-up showed no significant difference in neurodevelopment, growth, and atopy-related outcomes at the corrected age of five to seven years in children who had participated as EP infants (SS versus TS probiotic) in the SiMPro trial. Previous follow-up studies reporting no effect of early postnatal exposure to probiotics on long-term neurodevelopment in preterm infants are reassuring in the context of our results [43]. Follow-up of very preterm infants allocated to probiotic or placebo in the ProPrems trial (*n* = 1200) showed no significant difference in survival free from major neurodevelopmental impairment at age two to five years. The rate of deafness was reduced in probiotic recipients [43]. Two smaller RCTs assessing *L. reuteri* DSM 17938 supplementation have reported modest early improvement in language development but no effect on growth or general neurodevelopment at two years, despite improved head growth in the first month of life [44,45]. In a quasi-experimental study, including 233 very preterm infants (gestation < 32 weeks, birth weight < 1500 g), supplementation with *B. bifidum* NCDO 2203 and *L. acidophilus* NCDO 1748 from the first postnatal week until 34 weeks’, postmenstrual age significantly reduced neurodevelopmental impairment at 24 months of corrected age (RR 0.30, 95% CI 0.16–0.58) [46]. A recent Canadian multi-center cohort study (*n* = 5191 children born at <29 weeks’ gestation) reported that probiotic supplementation was safe and may confer modest benefit in neurodevelopment and growth. The propensity score-matched analyses suggested reduced risk of significant neurodevelopmental impairment in probiotic-supplemented versus non-supplemented infants [47]. Results of various meta-analyses suggest that probiotic supplementation significantly improved weight gain and reduced the risk of feeding intolerance and necrotizing enterocolitis during the neonatal intensive care unit stay. However, it did not appear to influence long-term growth or neurodevelopment [48,49,50].

Probiotics may have limited effects on paediatric neurobehavioral conditions. In a systematic review including seven RCTs, supplementation with *Lactobacillus rhamnosus* GG in the first six months of life was the only intervention that reduced later diagnoses of ADHD or Asperger syndrome [51]. The remaining six trials reported no cognitive benefits, underscoring the limitations of the current evidence on probiotic effects on neurodevelopmental conditions in children and adolescents [51]. Further research is justified considering the distinct gut microbiota profiles in children with ADHD and autism and the potential of probiotics to improve outcomes in such conditions [52,53,54,55,56].

Atopy-related outcomes were comparable between the SS and TS groups in our follow-up study. Earlier studies comparing probiotic versus placebo supplementation have reported no protective effects of PS against allergic rhinitis, asthma, eczema and /or food allergy in early years of life [57]. However, investigators have reported reduced risk of hospital admissions in early childhood due to respiratory and gastrointestinal illness following probiotic exposure in the neonatal period [58]. The reduced susceptibility to such adverse outcomes in early childhood in the probiotic group may relate to enhanced gut mucosal immunity (via increased secretory IgA and epithelial integrity), suppression of colonization by pathogens and translocation, and systemic immunomodulatory effects, including downregulation of Toll-like receptor and TNF-α pathways [57,58].

The strengths of our study include the robust design of the original SiMPro RCT, minimizing the influence of confounding, comprehensive clinical data, optimal school-age follow-up rates (SS: 89.2%; TS: 95%), and the novel comparison of SS versus TS probiotics in EP infants at high risk of adverse long-term outcomes. To our knowledge, this is perhaps the first prospective follow-up study reporting such long-term outcomes of children who had participated as EP infants in the SiMPro RCT comparing SS versus TS probiotics. Our study has significant limitations that need to be acknowledged. The modest sample size was not powered for neurodevelopmental outcomes. The attrition over prolonged follow-up may have reduced statistical power further to detect a small but clinically meaningful difference, particularly within behavioral and executive function domains, as the later data were available only for those with face-to-face follow-up. Although the original study was an RCT with well-balanced baseline characteristics and no significant differences in morbidity that could have influenced adverse developmental outcomes, multiple factors arising between the neonatal period and the five-year assessment may have affected neurodevelopmental results. In addition, the absence of a control arm precluded a meaningful comparison of long-term outcomes between probiotics and the placebo group. Furthermore, the study population was derived from a single region, which may limit generalizability.

## 5. Conclusions

In summary, the follow-up of the SiMPro RCT cohort at school age indicates that early postnatal probiotic supplementation was safe and did not adversely affect long-term neurodevelopment in EP infants. Neither the single-strain nor the triple-strain Bifidobacteria conferred a measurable neurodevelopmental advantage at school age, reinforcing that probiotic choice in neonatal practice may be guided primarily by early clinical benefits rather than long-term cognitive or behavioral effects. Future research should focus on large, multi-center trials incorporating standardized definitions for neurodevelopmental outcomes/impairments, integrating with microbiome and metabolome profiling, for robust assessment of the effects of probiotics on long-term growth, neurodevelopment, and other outcomes in EP infants.

## Figures and Tables

**Table 1:** Definitions of disability.

Overall Disability	Criteria
Mild (At least one criteria)	A cognitive score between one and two standard deviations (SD) below the test mean, ambulant cerebral palsy (GMFCS level I/II)
Moderate (At least one criteria)	A cognitive score between two and three SD below the test mean, GMFCS level III CP (ambulant with aids), unilateral deafness
Severe (At least one criteria)	A cognitive score >3 SD below the test mean, GMFCS level IV/V CP, blindness (vision < 6/60), autism, bilateral deafness needing amplification

**Table 2:** Demographic characteristics of the original SiMPro RCT cohort.

		SS; *n* = 86	TS; *n* = 87	
		N (%)	N (%)	*p*
Sex	Female	38 (44.2%)	41 (47.1%)	0.698
	Male	48 (55.8%)	46 (52.9%)	
GA mean (SD)		25.96 (1.35)	25.79 (1.53)	0.434
Parental education * (*n* = 40, *missing n* = 133)				0.255
	secondary	4 (22.2%)	7 (31.8%)	
	post-secondary	3 (16.7%)	8 (36.4%)	
	tertiary	8 (44.4%)	4 (18.2%)	
	post-graduate	3 (16.7%)	3 (13.6%)	
SEIFA decile (*n =* 135) *				0.296
	1	4 (6.1%)	8 (11.1%)	
	2	7 (10.6%)	6 (8.3%)	
	3	5 (7.6%)	11 (15.3%)	
	4	5 (7.6%)	2 (2.8%)	
	5	12 (18.2%)	7 (9.7%)	
	6	5 (7.6%)	5 (6.9%)	
	7	3 (4.5%)	3 (4.2%)	
	8	13 (19.7%)	9 (12.5%)	
	9	3 (4.5%)	10 (13.9%)	
	10	9 (13.6%)	11 (15.3%)	
SEIFA tertile *				0.285
	1	19 (29.2%)	25 (35.7%)	
	2	26 (40.0%)	19 (27.1%)	
	3	20 (30.8%)	26 (37.1%)	

*: For all age follow-ups, including five-year-old corrected age follow-up. Abbreviations: GA: gestational age, SD: standard deviation, SEIFA: socio-economic indexes for areas, SS: single strain, TS: triple strain.

**Table 3:** Neurodevelopmental and neurobehavioral outcomes at five years.

Outcome	SS (*n* = 66)	TS (*n* = 74)	*p*
Any CP	4 (6.1%)	4 (5.4%)	1.000
CP severity (GMFCS) 4–5	2 (50.0%)	3 (75.0%)	1.000
2–3	2 (50.0%)	1 (25.0%)	
1	0 (0%)	0 (0%)	
Autism			
suspected	2 (3.0%)	6 (8.1%)	0.271
confirmed	2 (3.0%)	6 (8.1%)	0.135
Autism severity level 2	1 (25%)	1 (20.0%)	1.000
Autism severity level 3	3 (75%)	4 (80.0%)	
Blindness	0 (0.0%)	0 (0.0%)	-
Deafness	1 (1.5%)	0 (0.0%)	1.000
Completed WPPSI	56 (85%)	56 (76%)	0.226
WPPSI-IV age (yr) median (IQR)	5.25 (5.25, 5.33)	5.25 (5.25, 5.33)	0.816
WPPSI-IV FSIQ median (IQR)	84 (127, 98)	98 (84, 105)	0.369
SDQ completed ^$^	15/ 56 (23%)	16/56 (28.6%)	0.998
SDQ scores: Median (IQR)			
Total difficulties	9 (7, 15)	15 (8.5, 17.5)	0.132
Emotional symptoms	1 (0, 4)	2 (0, 4)	0.576
Conduct problems	1 (0, 3)	3 (0.5, 4)	0.295
Hyperactive inattention	6 (2, 8)	6 (5, 10)	0.176
Peer relationship problems	1 (0, 3)	2 (0, 3.5)	0.737
Prosocial behavior	9 (7, 9)	6 (4, 9)	0.132
Impact	0 (0, 2)	0 (0, 1)	0.655
BRIEF completed ^$^	26/56 (46.4%)	34/56 (60.7%)	0.317
**BRIEF T-scores: Median (IQR)**			
Inhibit scale	52 (45.75, 67.25)	60.5 (51, 74)	0.139
Emotional control scale	50.5 (43, 64.5)	57 (45, 77)	0.324
Shift scale	48 (42.75, 62)	52 (42, 63)	0.520
Working memory scale	59 (49.5, 76.25)	65 (54, 83)	0.170
Plan/organize scale	58 (46.25, 64.25)	64 (51, 75)	0.129
Inhibitory self-control index	52.5 (43.75, 70.25)	60 (49, 77)	0.191
Flexibility index	47.5 (41.75, 66.75)	59.5 (44, 70)	0.366
Emergent metacognition index	58 (47, 75)	66 (53, 82)	0.161
Global executive composite	54 (46.5, 68.5)	61 (51, 81)	0.143

^$^: SDQ and BRIEF–P were completed only for the face-to-face assessments. Abbreviations: BRIEF–P: Behavior Rating Inventory of Executive Function–Preschool, CP: Cerebral Palsy, GMFCS: Gross Motor Functional Classification System, SDQ: Strengths and Difficulties Questionnaire, and WPPSI: Wechsler Preschool and Primary Scale of Intelligence

**Table 4:** Overall disability at school age (five years).

Overall Disability Category	SS (*n* = 66)	TS (*n* = 74)	OR (95% CI)	*p*	aOR * (95% CI)	*p*
Normal	44 (66.7%)	41 (55.4%)	1.00		1.00	
Mild	12 (18.2%)	16 (21.6%)	1.43 (0.61, 3.39)	0.415	1.39 (0.57, 3.34)	0.468
Moderate-severe	10 (15.2%)	17 (23.0%)	1.82 (0.75, 4.44)	0.185	1.77 (0.71, 4.39)	0.218

* Adjusted for SEIFA centiles.

**Table 5:** Growth parameters at school age (five years).

Outcome	Total *n*	SS Median (IQR)	TS Median (IQR)	*p*
Height (cm)	107	108.0 (105.0, 110.8)	108.0 (105.25, 112.10)	0.701
Height z score	107	−0.05 (−0.68, 0.54)	−0.05 (−0.63, 0.81)	0.707
Weight (kg)	107	17.55 (16.16, 19.65)	17.70 (15.85, 18.95)	0.297
Weight z score	107	−0.13 (−0.64, 0.63)	−0.08 (−0.75, 0.37)	0.707
HC (cm)	107	50.00 (49.50, 51.63)	50.30 (49.10, 51.55)	0.857
HC z score	107	−0.27 (−0.56, 0.65)	−0.10 (−0.78, 0.61)	0.707

**Table 6:** BP, BMI (at five years), and atopy-related outcomes (six years).

	SS (*n* = 74)	TS (*n* = 79)	
	N (%)	N (%)	*p*
^@^SBP *Median (IQR)*	103 (98, 107)	103 (97.5, 107)	0.948
^@^DBP *Median (IQR)*	65 (61, 69)	64 (60.5, 68)	0.414
^@^BMI *Median (IQR)*	15.14 (14.24, 16.20)	14.89 (13.95, 15.79)	0.220
^@^BMI z-score *Median (IQR)*	−0.06 (−0.61, 0.58)	−0.21 (−0.78, 0.34)	0.220
**Completed ISAAC questionnaire**	37 (50.0%)	41 (51.9%)	0.826
(1) Wheeze			
Diagnosed ever	10 (27.0%)	12 (29.3%)	0.478
Wheezing in last 12 m	5 (13.5%)	8 (19.5%)	0.553
(2) Asthma			
Diagnosed ever	0 (0.0%)	4 (9.8%)	0.090
Wheeze episodes last 12 m	6 (16.2%)	8 (19.5%)	0.705
Frequency of wheeze episodes in last 12 m			0.424
1	2 (5.4%)	1 (2.4%)	
2	3 (8.1%)	7 (17.1%)	
3	1 (2.7%)	0 (0.0%)	
(3) Rhinitis			
Diagnosed ever	3 (8.1%)	2 (4.9%)	0.664
episodes in last 12 m	12 (32.4%)	9 (22.0%)	0.297
(4) Hay fever			
Diagnosed ever	12 (32.4%)	12 (29.3%)	0.762
episodes in last 12 m	5 (13.5%)	10 (24.4%)	0.262
(5) Eczema (atopic dermatitis)			
Diagnosed ever	15 (40.5%)	15 (36.6%)	0.720
episodes in last 12 m	8 (21.6%)	13 (31.7%)	0.316

^@^: SBP, DBP, BMI measured only for face-to-face assessments; ISAAC was offered to all eligible for follow-up. Abbreviations: BMI: Body Mass Index, DBP: diastolic blood pressure, and SBP: systolic blood pressure.

## Data Availability

The original contributions presented in this study are included in the article. Further inquiries can be directed to the corresponding authors.

## Abbreviations

The following abbreviations are used in this manuscript: aOR: adjusted Odd’s Ratio for SEIFA; BRIEF–P: Behavior Rating Inventory of Executive Function—Preschool version (BRIEF–P); CP: Cerebral palsy; ISAAC: International Study of Asthma and Allergies in Childhood; GMFCS: Gross Motor Function Classification System; HC: head circumference; OR: Odd’s Ratio; PS: probiotic supplementation; RCT: Randomized Controlled Trial/s; SEIFA: Socio Economic Indexes for Areas; SDQ: Strengths and Difficulties Questionnaire; SS: Single strain, TS: Triple-strain; WPPSI: Wechsler Preschool and Primary Scale of Intelligence.
